# Sentinel fall presenting to the emergency department (SeFallED) – protocol of a complex study including long-term observation of functional trajectories after a fall, exploration of specific fall risk factors, and patients’ views on falls prevention

**DOI:** 10.1186/s12877-022-03261-7

**Published:** 2022-07-18

**Authors:** Tim Stuckenschneider, Jessica Koschate, Ellen Dunker, Nadja Reeck, Michel Hackbarth, Sandra Hellmers, Robert Kwiecien, Sandra Lau, Anna Levke Brütt, Andreas Hein, Tania Zieschang

**Affiliations:** 1 Department for Health Services Research, Geriatric Medicine, School of Medicine and Health Sciences, Carl Von Ossietzky University, Ammerländer Heerstraße 114-118, 26129 Oldenburg, Germany; 2 Department of Health Services Research, Junior Research Group for Rehabilitation Sciences, School of Medicine and Health Sciences, University of Oldenburg, Oldenburg, Germany; 3 Department for Health Assistance Systems and Medical Device Technology, Services Research, School of Medicine and Health Sciences, Carl Von Ossietzky University, Oldenburg, Germany; 4 Institute of Biostatistics and Clinical Research, University of Muenster, Münster, Germany

**Keywords:** Falls prevention, Emergency department, Older adults, Machine learning, Patient involvement, Dynamic balance, Perturbation, Aerobic fitness, Activities of daily living, Cognitive impairment

## Abstract

**Background:**

Falls are a leading cause for emergency department (ED) visits in older adults. As a fall is associated with a high risk of functional decline and further falls and many falls do not receive medical attention, the ED is ideal to initiate secondary prevention, an opportunity generally not taken. Data on trajectories to identify patients, who would profit the most form early intervention and to examine the impact of a fall event, are lacking. To tailor interventions to the individual’s needs and preferences, and to address the whole scope of fall risks, we developed this longitudinal study using an extensive assessment battery including dynamic balance and aerobic fitness, but also sensor-based data. Additionally, participative research will contribute valuable qualitative data, and machine learning will be used to identify trips, slips, and falls in sensor data during daily life.

**Methods:**

This is a mixed-methods study consisting of four parts: (1) an observational prospective study, (2) a randomized controlled trial (RCT) to explore whether a diagnostic to measure reactive dynamic balance influences fall risk, (3) machine learning approaches and (4) a qualitative study to explore patients’ and their caregivers’ views. We will target a sample size of 450 adults of 60 years and older, who presented to the ED of the Klinikum Oldenburg after a fall and are not hospitalized. The participants will be followed up over 24 months (within four weeks after the ED, after 6, 12 and 24 months). We will assess functional abilities, fall risk factors, participation, quality of life, falls incidence, and physical activity using validated instruments, including sensor-data. Additionally, two thirds of the patients will undergo intensive testing in the gait laboratory and 72 participants will partake in focus group interviews.

**Discussion:**

The results of the SeFallED study will be used to identify risk factors with high predictive value for functional outcome after a sentinel fall. This will help to (1) establish a protocol adapted to the situation in the ED to identify patients at risk and (2) to initiate an appropriate care pathway, which will be developed based on the results of this study.

**Trial registration:**

DRKS (Deutsches Register für klinische Studien, DRKS00025949). Prospectively registered on 4^th^ November, 2021.

**Supplementary Information:**

The online version contains supplementary material available at 10.1186/s12877-022-03261-7.

## Background

Falls are a main reason for emergency department (ED) visits in older adults [1]. Around 33% of community-dwelling older adults fall at least once a year with many of them becoming recurrent fallers [2, 3]. Falls diminish quality of life and increase mortality and morbidity [4]. As fall incidence and severity rise with age, falls will continue to be a burden for healthcare systems worldwide, especially in the context of the demographic change [3, 5].

Numerous risk factors for falls have been identified such as age, sex, cognitive decline, vertigo, muscle weakness, (dynamic) balance dysfunction, or polypharmacy [6–9]. Consequences of falls include physical as well as psychosocial sequelae. Physically, about 14% of falls lead to fractures, and around 10% of falls result in traumatic head injuries [10, 11]. Psychosocially, falls may cause fear of falling, which is a relevant fall risk factor itself, with prevalence ranging between 30 to 73% [12, 13]. Fear of falling may result in reduced physical activity [14], and affect social participation, thus, increasing the risk of social isolation and loss of independence [15, 16]. Therefore, the manifold consequences of falls do not only impair quality of life, but also increase the risk of further diseases and disorders such as depression and dementia [16–18], potentially forming a vicious circle.

Individuals presenting to the ED with a fall, who are directly discharged, are an easy to identify, but yet understudied high-risk group for further falls and functional decline [19, 20]. To optimize patient care, it is crucial to initiate secondary prevention programs for these individuals as they seek medical attention in the ED. Long term observation of this group may enable the identification of different functional trajectories [20, 21], thereby providing a solid basis for well-founded decision-making, and tailored falls prevention interventions in the future.

To identify functional trajectories, a comprehensive geriatric assessment is needed, whose informative value can be improved by modern sensor technology. Further, an in-depth analysis of fall risk factors is mandatory. As dynamic reactive balance and aerobic fitness are not only discussed to be relevant fall risk factors but may also present treatment targets for future trials, they should be included in such an assessment battery. Dynamic balance dysfunctions are known to increase fall risk [7–9]. However, previous research indicates that even a single perturbation-based dynamic balance session influences functional trajectories and fall risk positively [22]. Therefore, observational studies using perturbation to assess dynamic balance have to control for potentially confounding effects of such a diagnostic session. The role of aerobic fitness as a risk factor is less clear and its association with static balance mostly based on cross-sectional findings [23, 24], making it a promising research target for longitudinal studies.

The worrying sequelae of falls for the individual but also their societal impact, call for action to strengthen and establish effective prevention programs, promoting high compliance of the participants [25]. Here, it is essential to take the populations’ specific needs into account. Patient-centered approaches have gained importance to identify potential barriers and facilitators to ensure adherence to such programs [26]. Therefore, exploring patients’ and their caregivers’ views on falls prevention seems crucial to build a realistic foundation for tailored intervention programs [27, 28].

The SeFallED study aims to identify long-term trajectories of older adults, presenting to the ED without hospital admission, after a sentinel fall. The study will combine geriatric assessments, sensor data and machine learning approaches. Further, the prognostic value of different, so far rarely studied risk factors such as aerobic fitness and dynamic reactive balance in predicting the trajectories, while controlling for potentially confounding effects of the respective risk factor assessments will be analyzed. Finally, these results will be combined with information on individual barriers, preferences and needs from the patients’ and caregivers’ perspectives to select and evolve adequate fall prevention programs in the future.

## Methods/design

### Study design

This study protocol describes a mixed-methods study, consisting of four parts: (1) an observational prospective study to identify different trajectories of performance on activities of daily living, (2) a randomized controlled trial using a parallel group design (1:1) to explore whether a diagnostic to measure reactive dynamic balance influences fall risk, (3) machine learning a) to record data using inertial measurement units (IMU) and (depth-) cameras of human reactions to standardized perturbations in order to train classifiers and b) to identify slips, trips, and falls from IMU sensor data in real life, and (4) a qualitative study to explore patients’ and their caregivers’ views on falls prevention to identify facilitators and barriers for future interventions [29]. A patient advisory board consisting of older adults that have experienced fall(s) will accompany this study.

**Fig. 1:**
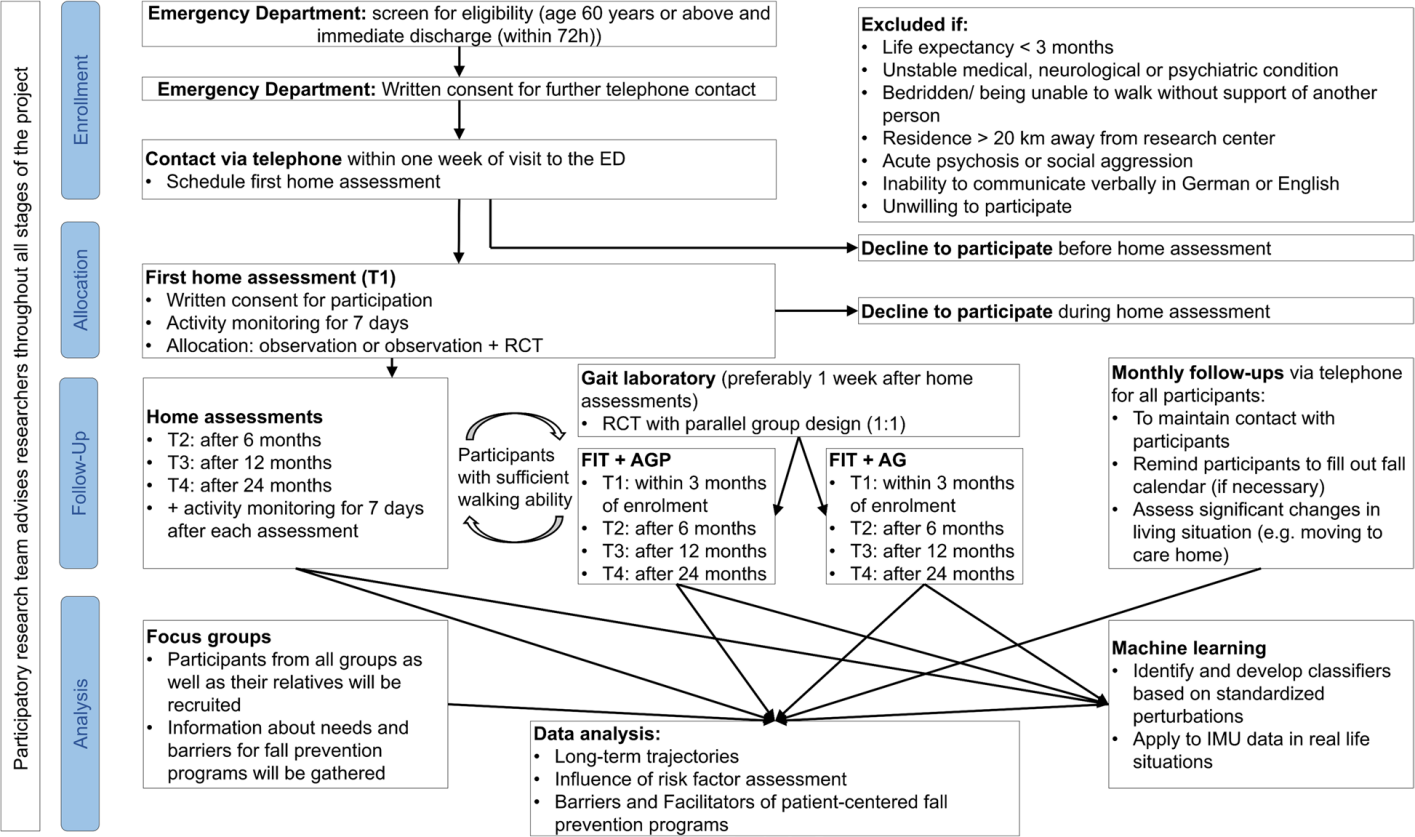
Flow diagram for the study design (FIT = participants with sufficient walking ability; FIT + AGP Aerobic fitness, Gait analysis and Perturbation based dynamic balance; FIT + AG = Aerobic fitness and Gait analysis)

The study will be conducted at the Carl von Ossietzky University in Oldenburg, Germany from October 2021 until January 2024, in accordance with the Declaration of Helsinki and was approved by the Medical Ethics Committee of the University of Oldenburg (number 2021 – 120). All participants will provide written informed consent to participate in the study. The consent forms are designed to be signed by either the participant or if needed by the participant and his / her legal guardian or family member. Ethical guidelines for research conducted with adults that lack the capacity to give consent will be followed and include the principle of group, the subsidiarity principle and the minimal risk standard [30]. Further, study information has been adapted to facilitate the participant’s understanding of the study, which will be provided in addition to the regular consent form, if necessary. Figure 1 shows the study design in a flow diagram.

### Participants and recruitment

It is the aim to recruit 450 older adults, who meet the following inclusion criteria: 1) age 60 years or above, 2) presented to the ED of the Klinikum Oldenburg after a fall and were discharged within 72 h, 3) informed consent.

Exclusion criteria are as follows: 1) life expectancy of less than 3 months, 2) unstable medical, neurological or psychiatric condition, 3) bedridden or being unable to walk without support of another person, 4) residence more than 20 km away from the research center, 5) acute psychosis or social aggression, 6) inability to communicate verbally in German or English. Furthermore, participants that 1) are completely dependent on walking aids 2) or subjectively not able to walk 400 m in less than 5 min will be included in the observational part of the study but deemed ineligible for visiting the gait laboratory.

A study nurse will be present at the ED from Monday to Friday during regular working hours and address all suitable individuals to obtain written consent for further contact. These individuals will be contacted within a week of their visit to the ED to schedule an appointment for the first home assessment during which written consent for participation will be provided. The staff in the ED will hand out flyers with relevant information for participation and contact data outside regular working hours of the study nurse. Recruitment will be ongoing between October 2021 and January 2023.

### Randomization and blinding

Two thirds of the 450 participants will take part in the tests in the gait laboratory. To control for the potential influence of assessing dynamic balance, these 300 participants will be randomly allocated to either a perturbation or no perturbation group using a 1:1 ratio block randomization. Randomization will be stratified in four different strata based on sex (male / female) and cognitive status (normal / impaired). We will stratify participants into cognitively normal and cognitively impaired using the result of the Montreal Cognitive Assessment (MoCA) at the first home visit. The chosen cutoff of ≤ 24 points increases sensitivity and specificity of the MoCA and reduces false positive results [31–33]. The randomization lists will be prepared by an independent statistician and will not be accessible by the persons enrolling the subjects into the study. The lists will be stored on a secured data server and protected by a password only known by the principal and co-investigators. Assessors of functional performance and falls risk factors will be blinded to allocation.

### Data assessment

Data will be collected at the ED, in participants’ homes and at the gait laboratory. Furthermore, IMUs to measure physical activity as well as gait parameters will be worn by the participants during daily life over 7 days at each time point (T1 – T4). After the initial assessment, the researchers maintain contact with the participants through monthly phone calls, as part of the prospective falls recording in addition to fall calendars (Fig. 1). Within four weeks after presenting to the ED, participants will be visited at home for the first functional assessment (T1), which will be repeated after 6 (T2), 12 (T3) and 24 (T4) months. Besides the data collected during the home assessments, eligible participants will attend testing in the university’s gait laboratory. This appointment will preferably be scheduled a week after the respective home assessments.

### Outcome measures

#### Emergency department

All older patients in the ED are regularly assessed with a geriatric screening ‘Geriatrisches Screening bei Klinikaufnahme’ (Supplementary file 1), which consists of six yes / no questions with a higher score predicting a poor outcome and the need for co-management or treatment by a geriatric team. Furthermore, all participants will be assessed for delirium using the Confusion Assessment Method (CAM) for Intermediate Care (CAM-IMC), which has shown better sensitivity and specificity in comparison to the widely used CAM for Intensive Care Unit (CAM-ICU) and includes the Richmond Agitation-Sedation Scale [34–36]. From the chart data chronic diseases and medication will be extracted to complement information given by the participant as soon as consent is provided. In addition, routinely drawn blood samples will be stored for further analyses of biomarkers.

#### Home assessment

After providing written consent to participate in the study, participants’ characteristics (age, sex, education, number and name of medications, pre-existing illnesses including joint replacements, smoking status, alcohol consumption, the use of hearing, seeing, and walking aids, and the living situation (e.g., alone, in a care facility) will be assessed. Additionally, the participants will be asked for detailed information on the recent fall that led to the visit at the ED, including time of the fall, location, and activity before falling, direction of the fall, and injuries. For a comprehensive geriatric assessment an extensive assessment battery will be used consisting of standardized, validated and widely used instruments. A detailed overview is given in Table 1.

**Table 1:** Assessment battery during the home visits

Assessment tool	Outcomes	Timepoint
1. Participants’ characteristics (including personal and medical history)	Age, sex, education, pre-existing illnesses, usage of hearing, seeing and walking aids, living situation, smoking status, alcohol consumption, medication, joint replacements	T1
2. Specific fall history	Time of fall, location, activity before falling, direction of the fall, injuries	T1
3. German short falls efficacy scale (short FES-I) [37, 38]	Total score (ranging from 7 – 28)	T1, T2, T3, T4
4. Montreal Cognitive Assessment (MoCA) [39] a.MoCA memory index score (MoCA-MIS) [39, 40]	Total score (ranging from 0 – 30) Total score (ranging from 0 – 15)	T1, T2, T3, T4
5. Trail Making Test A and B (TMT A + B) [41]	Duration until completion in seconds & number of mistakes	T1, T2, T3, T4
6. (instrumental) activities of daily living: a.Lawton’s and Brody’s Index [42] b.Barthel Index [43] c.Jonkman Index [21]	Total score (ranging from 0 – 8) Total score (ranging from 0 – 100) Total score (ranging from 0 – 18)	T1, T2, T3, T4
7. Longitudinal Urban Cohort Ageing Study (LUCAS—FI) Functional Ability Index [44]	Functional ability classified in: Robust, postRobust, preFrail, Frail	T1, T2, T3, T4
8. Physical activity: a.German-Physical-Activity-Questionnaire 50 + (PAQ-50 +) [45] b.Physical Activity Scale for the Elderly (PASE) [46] c.Activity monitor (activPAL^©^) worn for 7 days	Energy expenditure per week Total score (ranging from 0 – 793) Number of steps, total active / inactive time	T1, T2, T3, T4
9. German Life Space Questionnaire (LSA-D) [47, 48]	Total score (ranging from 0 – 120)	T1, T2, T3, T4
10. Depressive Symptoms a.Depression in Old Age Scale (DIA-S) [49] b.Cornell Depression Scale [50]^a^	Total score (ranging from 0 – 10) Total score (ranging from 0 – 38)	T1, T2, T3, T4
11. Health-related quality of life EQ-5D – 3L + Scale [51, 52]	Total score (ranging from 0 – 15)	T1, T2, T3, T4
12. Functional performance: a.Hand grip strength test [53] b.Single leg stance test [54] ^b^ c.Short Physical Performance Battery Test (SPPB) [55]^b^	Grip strength measured in kg Duration in seconds Total score (ranging from 0 – 10)	T1, T2, T3, T4
13. Fall Calendar	Total number of falls during the follow up period	T2, T3, T4

^a^ The Cornell Depression Scale will be used instead of the DIA-S in case of severe cognitive impairment (MoCA < 18) or an existing diagnosis of dementia, ^b^ During the functional assessments, participants will be equipped with three wirelessly synchronized inertial measurement units (IMUs; Opal V1, Mobility Lab™ (ML), APDM, Inc., Portland, OR, USA), which will objectively assess postural sway and gait characteristics [56, 57]

#### Gait Laboratory (RCT for effect of perturbation protocol)

**Fig. 2:**
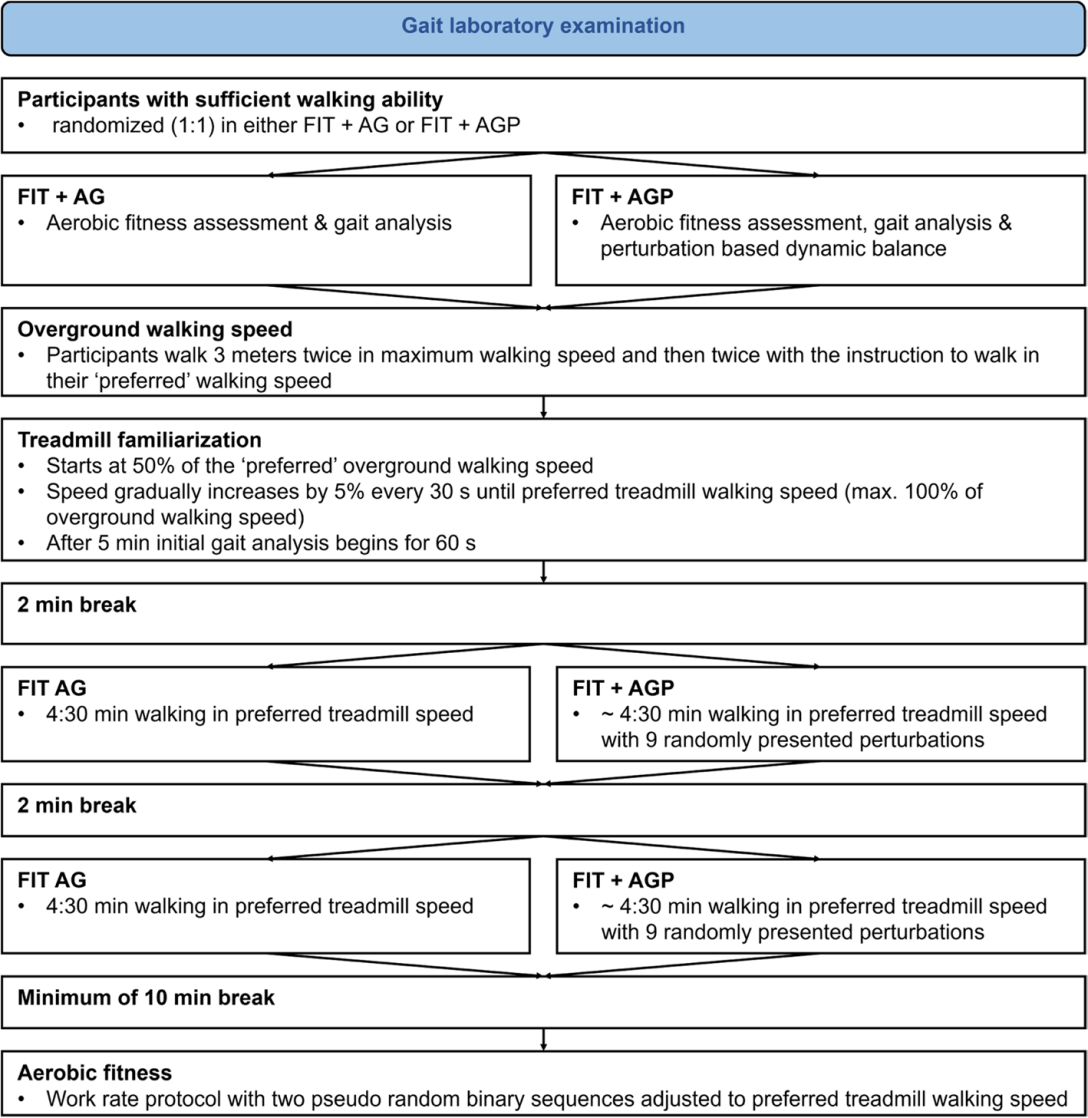
Gait laboratory examinations including the randomized controlled trial

Participants will visit the gait laboratory ideally one week after their home assessments. The time between fall and visit to the gait laboratory will be documented. At the lab participants will be equipped with an activity monitor (activPAL^©^, PAL Technologies Ltd, Glasgow UK) and six wirelessly synchronized IMU’s (Opal V1, Mobility Lab™ (ML), APDM, Inc., Portland, OR, USA) to measure gait characteristics during all the assessments in the laboratory. A flow chart of the assessments, conducted in the gait laboratory, is provided in Fig. 2.

To determine overground walking speed, which will be used for all further assessments at the gait laboratory, participants will walk 3 m twice with the instruction to walk as fast as they can without running, then twice with the instruction to walk with their preferred walking speed. The mean time of the two trials under the same instructions will be taken and used to calculate maximal and preferred overground walking speed in km/h, respectively. To minimize the effect of starting, stopping or turning, 1 m will be added at each end of the walk way.

#### Reactive dynamic balance

Reactive dynamic balance will be assessed on the M-Gait treadmill (Motek Medical B.V., Amsterdam, the Netherlands), which enables anterior–posterior, medio-lateral and pitch perturbations. The integrated split-belt of the treadmill with separate force plates allows to perturb either both legs or just one leg during walking. The participants are secured using a safety harness.

Familiarization on the treadmill will start at 50% of the preferred overground walking speed. Treadmill walking speed will be gradually increased by 5% every 30 s until the participants will reach either 100% of the previously measured overground walking speed or an individually more preferable walking speed. Familiarization will last 6 min, which is regarded to be sufficient according to previous research [58–60]. The final minute of the familiarization will be used to analyze the participants’ gait at the preferred gait speed with the aforementioned sensors (activPAL^©^, APDMs). Additionally, three Microsoft Kinect Azure cameras (Microsoft, USA) will be used as a motion capture system [61].

After a two-minute break, participants randomized to the *no perturbation group* will walk twice for 4 min and 30 s with the individually determined treadmill walking speed. Participants will have a two-minute break between the two trials.

Participants randomized to the *perturbation group* will complete a perturbation protocol with similar duration and break time. The perturbation protocol will start with 30 s of the preferred treadmill walking speed, followed by 9 perturbations randomly presented to the participants. Time between perturbations will vary between 20–30 s, which is deemed a sufficient washout period [62]. Perturbations will be triggered through the integrated force plates at initial foot contact through custom application (D-Flow version 3.34.2, Motek Medical BV, Amsterdam, The Netherlands). Using the split belt option, each leg will be perturbed in anterior–posterior direction twice, mimicking slips and trips. Slips will be simulated by an acceleration of 3 m∙s^−2^ to a maximum of 180% of treadmill walking speed for 0.42 s. Trips will be provoked similarly with a deceleration of 3 m∙s^−2^ to a minimum of 40% of treadmill walking speed, which is in line with previous research [58, 59, 63, 64]. Besides these single leg perturbations, an emergency stop as in public transport will be imitated with both belts decelerating by 9 m∙s^−2^ for 0.12 s [58, 65]. Further, the protocol will include two contralateral sways, perturbing each leg once by a 5-cm platform translation in 3 m∙s^−2^ [58, 59]. A pitch of + and—5° respectively, with a duration of 1.0 s will imitate small slope changes as present on sidewalks or at bus stops. Gait adaptations to these perturbations will be measured throughout the trials, using the aforementioned systems.

#### Aerobic fitness

**Fig. 3:**
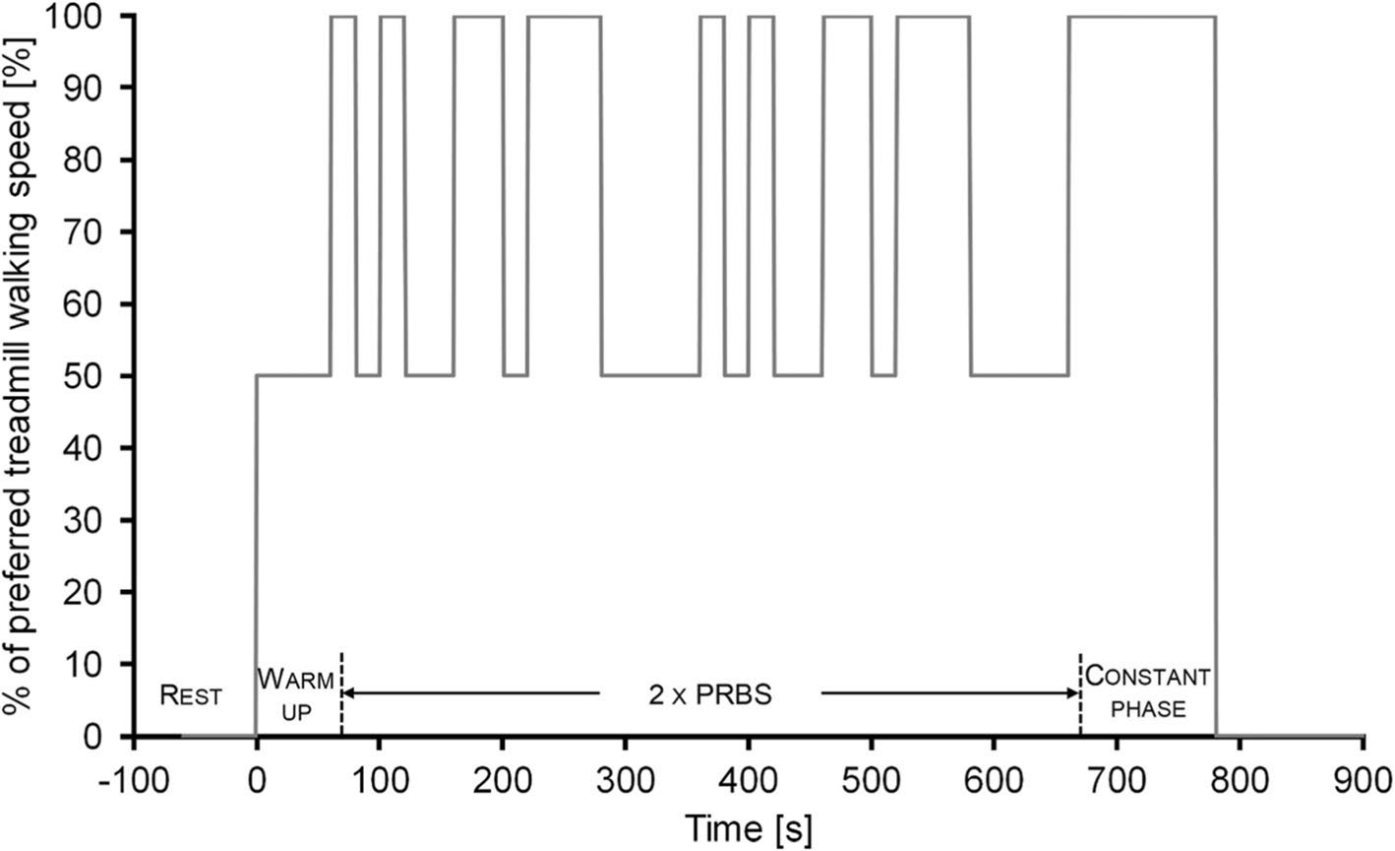
Description of the work rate protocol, encompassing a warm up phase, two pseudo random binary sequences (PRBS) and a constant work rate phase on the treadmill

All participants will complete a treadmill test, assessing cardiorespiratory kinetics as an indicator of aerobic fitness [66]. Participants will be equipped with a face mask connected to a mobile system (MetaMax3B, Cortex Biophysik GmbH, Leipzig, Germany) to measure breath-by-breath gas exchange and will wear an ECG belt (CustoGuard belt 3, Customed, Ottobrunn, Germany) to assess beat-to-beat heart rate. The work rate protocol on the treadmill consists of two pseudo random binary sequences [67–69] which will change between 50 and 100% of the preferred gait speed, as assessed during familiarization on the treadmill. The protocol to assess aerobic fitness is shown in Fig. 3.

### Focus groups

In order to explore patients’ preferences, needs, facilitators and barriers of participating and adhering to falls prevention interventions and to support a patient centered intervention, focus group interviews will be established as part of the project [70]. 72 persons consisting of participants and caregivers will be recruited to join twelve focus groups (k = 6 with patients, k = 6 with caregivers).

Audio data from the focus group interviews will be transcribed verbatim with two multidisciplinary independent researchers analyzing the data. Content analysis according to Kuckartz will be applied with categories such as needs, preferences, barriers and facilitators deductively derived from the Theoretical Domains Framework [71]. Furthermore, categories can be derived inductively from the material recorded [72].

### Sensor data acquisition and machine learning

Normal and reactive individual gait patterns are recorded via both, the two activity sensor systems (Inertial Measurement Unit consisting of accelerometer, gyroscopes, and magnetometers—activPAL^©^, APDM) and the three front-facing depth cameras (Kinect Azure). The camera data are used to identify the spatial–temporal gait parameters and to label the perturbations and the associated reaction strategy. Based on this data, the individual reaction of a subject is identified using the IMU data of the two sensor systems (activPAL^©^, APDM).

Depending on the degree of homogeneity of the reaction pattern, either general / inter-individual classifiers (high degree of uniformity) can be trained or individual classifiers (high individual characteristics) can be constructed. The derived classifiers will be used to recognize slip, trip, and fall patterns over the time in IMU data during daily life.

### Safety

All serious adverse events / adverse events will be recorded on study specific adverse event forms. Overexertion during gait laboratory examinations will be avoided by using the preferred gait of the individual participant on the treadmill. Furthermore, breaks can be extended as needed and participants will be asked regularly if they feel fit to continue. If subjective or objective signs of fatigue or overexertion are seen, the protocol will be immediately interrupted. All adverse events will be reported to the principal investigator.

### Sample size

The recruitment goal of 450 older adults for the observational part of the study is the result of an analysis of the hospital data base of the Klinikum Oldenburg from the year 2018, which revealed a total of 1.182 patients, presenting to the ED without admission, aged between 60 and 103 years. These patients were registered within the symptom-groups ‘problem of the extremities’, ‘head trauma’ or ‘fall’. We assume that 48% of these patients are ineligible due to in- and exclusion criteria as published by Barker and colleagues and expect a further drop out of 38% due to not giving consent for study participation [73].

An independent biostatistician calculated the sample size for the embedded randomized controlled trial (RCT). Sample size calculation was performed via computer simulations based on the study of Pai and colleagues [22]. The level of significance was set as α = 0.05. As we aim for a power of 80% a sample size of at least *N* = 245 is required. According to previous research, a dropout of 17% may be expected in this subgroup, which led to the decision to aim for 300 participants [73].

### Statistical analysis

The endpoints will be compared between different home assessments: T1 (within 4 weeks), T2 (6 months), T3 (12 months), and the following yearly examinations after ED attendance. The association between multiple variables (e.g. age, time since recruitment etc.) and the trajectories of the different endpoints will be analyzed fitting a linear mixed model (LMM) / generalized linear mixed models, (GLMM, i. e. Logistic, Negative Binomial, Ordinal). For metric endpoints, we will check the normal distribution of the residuals in the corresponding LMM via Lilliefors-Test or descriptively via Skewness. We also plan to analyze stratified models (stratified e.g. by sex and / or initial physical fitness). Regarding the trajectories in the entire group, we will use the Friedman test for metric / ordinal data, if normal distribution of the residuals is not valid. The data obtained during the gait lab visit (gait analysis, aerobic capacity, physical activity) will be compared in a similar fashion. To identify simple predictors of declines in functional performance and physical activity as well as fall risks, that potentially can be obtained directly in the ED or at the general practitioner, regression analysis will be used. Setting parameters of the gait analysis and the cardiorespiratory fitness test as dependent variable and parameters of the home assessments as independent parameters, predictive factors for fall risks will be analyzed. Local significance level will be set to 5%. We will deal with missing value problems via complete Likelihood approach under the missing at random-assumption (MAR) in parametric models.

For the included RCT, we will analyze if a diagnostic perturbation session influences prospective fall risk. We will fit a logistic regression model, and will calculate p-values for coefficients plus estimates of the coefficients plus 95% confidence intervals.

Statistical analyses will be performed according to the ICH Guideline E9 ‘Statistical Principles for Clinical trials’ using validated statistical software (SAS, SPSS, R).

### Data management

Data will be managed using unique study codes provided by an independent statistician. These will be used to code and file all electronic information that will be stored on secured university systems. Paper and pencil tests will be stored in a cabinet with a lock. Two different study nurses will enter the data collected from paper and pencil tests into a custom-made Research Electronic Data Capture (REDCap) database hosted at the Carl von Ossietzky University Oldenburg. REDCap is a secure, web-based software platform, designed to support data capture for research studies [74, 75].

### Patient and public involvement

A participatory research team (PRT) will be involved as consultants, which was approved by the Medical Ethics Committee of the University of Oldenburg (2021 – 106). The PRT consists of six older adults (66 to 84 years old), who have experienced falls in the past. The group is heterogeneous with regard to sex, age, school education, living situation, and the use of mobility aids.

Approximately 30 meetings between researchers and the PRT will take place throughout the course of the SeFallED study. The PRT will provide feedback for example to the comprehensibility of consent forms, the burden of the assessment batteries, strategies of participant recruitment and the guidelines for the focus group interviews. Further, it is planned to engage the PRT in conducting and analyzing the focus group interviews. All meetings between the PRT and the researchers will be documented with focus on the remarks of the PRT. The PRT’s feedback, and whether and to what extent it will lead to changes in study procedures, will be documented [76, 77]. In addition, researchers and the PRT will fill in the Public and Patient Engagement Evaluation Tool every six months [76, 77]. Members of the PRT will receive an expense allowance of 20€ per hour according to documented working hours, which include meetings and working tasks between meetings.

## Discussion

As falls are a major public health problem, the World Health Organization called for effective intervention strategies in their report ‘Step Safely’, published in 2021 [78]. The SeFallED study will aid in developing such strategies by systematically assessing the risk of older adults who presented to the ED after a fall, a group at high risk for further falls [79].

One strength of the SeFallED study is its long observation period (> 12 months), which may be increased by further funding, with yearly follow-up visits and monthly telephone calls. While fall research guidelines demand a prospective data collection for at least up to 12 months [80], it is likely that long-term consequences of a fall may either last well beyond this period or may only manifest after a certain time has passed [81]. Our long-term data will provide better insight and deepen our understanding of the consequences of falls in older adults, which is needed to design tailored interventions and to inform healthcare professionals and policy makers.

Another strength of the SeFallED study is its mixed methods approach, which combines both qualitative and quantitative data and includes machine learning aspects. Whereas quantitative data is needed to calculate risk scores that may facilitate decision making processes in the future, the qualitative approach, involving patients’ representatives, may enhance appropriateness and relevance of the research, and hence the quality of the study [26, 82]. Especially the collaboration with a PRT has shown promise to benefit the research process as well as its outcomes [83]. The potential of smart systems to enhance care for older adults, but also to detect falls or gait insecurities has been acknowledged in previous research [84, 85]. Therefore, adding machine learning to the observational data of older individuals after a sentinel fall, may offer a broad spectrum of innovative approaches in fall risk assessment, but also falls prevention.

The extensive assessment battery with its considerable number of outcome measures will result in a robust data set, and can be considered an additional strength of the SeFallED study. Researchers will work together with the PRT to deliver strategies such as splitting up home assessments into two days in highly vulnerable participants to reduce the participants’ burden, and successfully implement the extensive assessment battery. Furthermore, data collection will be done in the participants’ homes, which may help to alleviate discomfort and stress experienced particularly in laboratory settings. Nevertheless, data collection at home bears the risk of unplanned disturbances affecting the participants’ concentration such as the phone or door bell ringing. Such events will be noted by the assessors and discussed with the principal investigator.

Successful recruitment and adherence are key factors to the success of the study with drop out during the follow-ups presenting a major threat. Therefore, we decided that a study nurse is present in the ED to get into personal contact with potential participants. During the home assessments, the assessors will pay attention to promote a good atmosphere and allow questions and breaks in between assessments. To increase personal contact, which is important to minimize attrition [86], monthly phone calls are planned as part of the project. Furthermore, Christmas and thank you cards will be sent to the participants. Recruitment may be particularly challenging due to the ongoing COVID-19 situation, in which older adults are advised to reduce social contacts. Even though vaccination is available to the target population, the study team will undertake specific COVID-19 measures to increase participation in the study such as wearing FFP2 masks during the assessments, keeping a distance and communicating the vaccination status of the assessors.

The results of the SeFallED study will be used to identify risk factors with high predictive value for functional outcome after a sentinel fall. This will help to (1) establish a protocol adapted to the situation in the ED to identify patients at risk and (2) to initiate an appropriate care pathway, which will be developed based on the results of this study. (3) We will aim to select or (re)design existing interventions and / or develop effective interventions for older adults incorporating novel methods tailored to individual needs, barriers and preferences to lift fall prevention and individual compliance to a new level. (4) Automated falls detection based on machine learning algorithms may be integrated in devices used during daily life such as smart phones or wrist worn activity trackers, and will potentially play a major role in fall related research as well as for patient security in the future.

## Supplementary Information

**Additional file 1: Supplementary file 1. **‘Geriatrisches Screening bei Klinikaufnahme‘; unofficial English translation of the German version.

## Data Availability

Not applicable.

## Abbreviations

AG: Aerobic fitness and gait analysis
AGP: Aerobic fitness, gait analysis, and perturbation based dynamic balance
CAM: Confusion assessment Method
DIA-S: Depression in old age scale
DRKS: Deutsches Register für klinische Studien
ED: Emergency department
FES-I: Falls efficacy scale
FIT: Participants with sufficient walking ability
GLMM: Generalized linear mixed models
ICU: Intensive care unit
IMC: Intermediate care
IMU: Inertial measurement unit
LMM: Linear mixed model
LSA-D: German life space questionnaire
LUCAS – FI: Longitudinal urban cohort ageing study functional ability index
MAR: Missing at random
MoCA: Montreal cognitive assessment
MoCA-MIS: Montreal cognitive assessment – memory index score
PRBS: Pseudo random binary sequences
PRT: Participatory research team
REDCap: Research electronic capture
RCT: Randomized controlled trial
SPPB: Short physical performance battery
TMT: Trail making test
