# Supplementary material for: Sentinel fall presenting to the emergency department (SeFallED) – protocol of a complex study including long-term observation of functional trajectories after a fall, exploration of specific fall risk factors, and patients’ views on falls prevention
Source: BMC Geriatr. 2022 Jul 18;22:594. doi: 10.1186/s12877-022-03261-7 (PMC9289928; doi:10.1186/s12877-022-03261-7)
Supplement: Supplementary file 1 — Additional file 1: Supplementary file 1. ‘Geriatrisches Screening bei Klinikaufnahme‘; unofficial English translation of the German version. [file 12877_2022_3261_MOESM1_ESM.docx]

Supplementary File 1: ‘Geriatrisches Screening bei Klinikaufnahme‘

This tool has been developed by a geriatric working group in Bavaria, Germany (gem. Bayerischem Fachprogramm Akutgeriatrie, AFGiB 2011). It is similar to internationally established tools such as the identification of seniors at risk (ISAR) score. The English version below is an unofficial translation of the German version.

**Geriatric Screening at hospital admission**

| Age | ≥ 80 years | - Yes | - No |
| --- | --- | --- | --- |
| **Reduced Mobility** | - Gait insecurities *or* - Walking aids *or* - Rollator/walker | - Yes | - No |
| **Care** | - Level of care affirmed by health care insurance companies *or* - Needs assistance during daily life | - Yes | - No |
| **Multimorbidity** | - Dementia *or* - Confusion *or* - Anxiety *or* - Depression *or* - Impaired vision *or* - Strong hearing impairment *or* - Sensory deficits | - Yes | - No |
|  | - Vertigo (acute or within the last 4 weeks) *or* - Falls (more than one over the past year) *or* - Polypharmacy (more than 5 medications) *or* - Chronic pain (at least for three months) | - Yes | - No |
|  | - Frailty *or* - Pressure sore (at least degree 1) *or* - Cachexia (e.g., BMI < 18.5) *or* - Incontinence (acute or chronic) *or* - Frequent hospital admissions (at least one hospital admission within last 3 months) | - Yes | - No |
|  | Total number of Yes-Responses |  |  |
